# Effects of sulfate amendment and different temperatures on microbial community structure and hydrocarbon degradation in petroleum-contaminated soils

**DOI:** 10.3389/fmicb.2026.1993442

**Published:** 2026-10-28

**Authors:** Jun-Di Wang

**Affiliations:** College of Chemistry and Chemical Engineering, Xi’an Shiyou University, Xi’an, China

**Keywords:** environmental temperature, microbial community, microbial remediation, petroleum-contaminated soil, sulfate

## Abstract

Environmental temperature fluctuations have substantial effects on sulfate-mediated anaerobic remediation of petroleum-contaminated soils, yet systematic investigations into the underlying mechanisms and temperature-dependent degradation signatures remain limited. In this study, hydrocarbon degradation in oxygen-limited microcosms and microbial community succession were characterized over successive remediation stages. Degradation efficiencies increased with increasing temperature across the tested thermal gradients (5 °C, 20 °C, and 40 °C). Compared with control groups, the total hydrocarbon removal efficiency was significantly greater in sulfate-amended groups. All temperature treatments resulted in a similar degradation pattern: rapid hydrocarbon removal during the initial phase, followed by gradual deceleration during later incubation stages. Functional prediction revealed enrichment of gene families involved in fatty acid metabolism, ABC-type transport systems, and stress-responsive sigma factors. High-throughput sequencing revealed that *Pseudomonadota*, *Actinomycetota*, *Bacillota*, and *Planctomycetota* were the dominant microbial taxa whose relative abundances fluctuated in response to temperature, sulfate amendment, and remediation duration. Collectively, these findings suggest that sulfate addition and incubation temperature jointly modulate hydrocarbon-degradation dynamics. Nevertheless, temperature, rather than sulfate amendment, was the primary factor structuring microbial-community composition.

## Introduction

With the development of the petroleum industry, soil contamination resulting from petroleum hydrocarbon leakage during exploitation, well repair, fracturing, and related operations has become a serious threat to environmental safety and has directly affected human health and socioeconomic development (Bora et al., 2025). Microbial remediation represents a cost-effective and efficient approach that generates little secondary pollution when treating organic pollutants, and its application in the remediation of petroleum-contaminated soil has been extensively studied (Mokrani et al., 2024). The oxygen levels in subsurface soil profiles are low, suggesting that anaerobic remediation is an important *in situ* strategy for treating deep petroleum-contaminated soils (Trusei et al., 2021). To enhance the anaerobic degradation capacity of indigenous microorganisms, biostimulation with exogeneous electron acceptors such as sulfate has become an important strategy for the remediation of petroleum-contaminated soil (Yang et al., 2021). However, the remediation effectiveness of sulfate-induced anaerobic degradation varies because of seasonal temperature fluctuations, which directly constrains the consistent application of relevant remediation technologies (Wartell et al., 2021).

The anaerobic degradation mechanisms employed by microorganisms directly influence the degradation characteristics of petroleum hydrocarbons. Zhang et al. (2019) reported that when sulfate, nitrate, ferric iron, and bicarbonate were employed as electron acceptors for the anaerobic degradation of petroleum hydrocarbons, the degradation efficiencies of n-alkanes after 100 days were 64.36%, 32.58%, 62.79%, and 54.40%, respectively, whereas those of PAHs were 44.59%, 34.65%, 45.16%, and 32.69%, respectively. Moreover, the results of kinetic analysis revealed that the degradation half-lives of n-alkanes under the four electron acceptor conditions were 49.51, 99.02, 53.73, and 66.65 days, whereas those of PAHs were 58.74, 80.60, 60.80, and 87.74 days, respectively. Thus, the degradation efficiencies of both n-alkanes and PAHs induced by sulfate were higher than those observed under other anaerobic degradation mechanisms.

Environmental temperature also plays a critical role in shaping the microbial degradation of petroleum hydrocarbons. Hazaimeh and Ahmed (2021) reported that when bacterial communities were subjected to naphthalene degradation, the final degradation efficiency reached 100% at 37 °C; however, at 30 °C, the final degradation efficiency was 94%, and at 20 °C, the value decreased to 76%. However, when the temperature exceeded 37 °C, a notable decrease in the relative abundance of the bacterial community was observed, accompanied by a sharp reduction in naphthalene degradation efficiency. Qin et al. (2019) explored the underlying mechanisms and revealed that at low temperatures, the viscosity of petroleum hydrocarbons increases, whereas their volatility and water solubility decrease, thus collectively reducing the efficiency of biodegradation (Siles and Margesin, 2018). At high temperatures, microbial nucleic acids, proteins, and enzymes may denature or become inactivated, thus severely impairing microbial growth, metabolism, and reproduction and thereby decreasing the capacity for petroleum hydrocarbon biodegradation (Wang et al., 2021). These findings suggest that temperature-induced changes may substantially modify the characteristics of sulfate-induced anaerobic degradation of petroleum hydrocarbons, consequently affecting the overall remediation efficiency for contaminated soil. Therefore, determining the variation patterns of sulfate-induced anaerobic degradation of petroleum hydrocarbons in soil under different environmental temperature regimes is necessary.

Microorganisms can degrade petroleum hydrocarbons only within a certain temperature range. As the temperature approaches the optimum temperature for degradation, the metabolic activity of these microorganisms is enhanced, yielding improved degradation performance, which is reflected by an increase in the relative abundance of the relevant microbial populations (Zainab et al., 2023). Conversely, excessively high or low temperatures can inhibit microbial activity; suppress metabolism, growth, and reproduction; and result in reduced population abundance (Morais et al., 2021). Therefore, changes in the structure of indigenous microbial communities and associated metabolic pathways during sulfate-induced anaerobic degradation of petroleum hydrocarbons under varying temperature conditions are likely key factors that explain the observed differences in degradation efficiency. Although numerous researchers have investigated the relationship between temperature and microbial activity, the successional patterns of indigenous microbial communities and the corresponding shifts in metabolic pathways during sulfate-induced anaerobic remediation of petroleum-contaminated soil remain uncertain.

In this study, soil collected from a petroleum exploitation area was spiked with crude oil and subjected to a series of laboratory-scale simulation experiments involving sulfate-mediated remediation of petroleum-contaminated soil in oxygen-limited microcosms under different environmental temperature conditions. The characteristics of sulfate-mediated degradation of petroleum hydrocarbons in oxygen-limited microcosms at various temperatures were evaluated on the basis of the degradation efficiency and biodegradation kinetics. Illumina MiSeq sequencing was subsequently employed to analyze the successional dynamics of indigenous microbial communities and the associated changes in metabolic pathways at different degradation stages. This study aimed to provide greater insights into the effects of temperature variation on sulfate-mediated biodegradation of petroleum hydrocarbons in contaminated soil and to elucidate the potential microbiological mechanisms underlying this process.

## Materials and methods

### Soil collection and bioremediation design

Soil was collected from an area uncontaminated by petroleum hydrocarbons in northern Shaanxi Province. The surface layer was removed, and soil below a depth of 100 cm was selected. Crude oil was also obtained from northern Shaanxi (Jieliang et al., 2023). The collected soil was sieved, and its basic physicochemical properties (details are shown in Table 1) were determined before being stored for later use.

**Table 1:** Physicochemical and biological properties of the soil.

Main characteristics	Values
Total petroleum hydrocarbon (mg/kg dry soil)	<500
Organic carbon (mg/kg)	2,800 ± 31.74
Moisture content (%)	4.8 ± 0.03
pH	8.1 ± 0.09
Total phosphorus (mg/kg)	479.26 ± 0.18
Available phosphorus (mg/kg)	15.92 ± 0.16
Total nitrogen (mg/kg)	1,063 ± 45.13

Appropriate amounts of nutrient solution (the macronutrient and salt solution contained the following: NaCl: 5 g; MgCl_2_·6H₂O: 2.5 g; NH_4_Cl: 20 g; KCl: 15 g; CaCl_2_·2H₂O: 0.25 g; and KH₂PO₄: 10 g; trace element solution A contained the following: HCl: 5 g; CoCl₂·6H₂O: 0.095 g; FeCl_2_: 0.75 g; MnCl_4_·4H₂O: 0.05 g; ZnCl_2_: 45 mg; H_3_BO_3_: 3 mg; Na₂MoO₄·2H₂O: 18 mg; NiCl₂·6H₂O: 12 mg; and CuCl_2_·2H₂O: 1 mg; trace element solution B contained the following: NaOH: 0.25 g; Na₂WO₄·2H₂O: 4 mg; and Na₂SeO_3_·5H₂O: 3 mg; the vitamin solution contained the following: CHN₇O_6_: 1 mg; C_6_H_5_NO_2_: 2.5 mg; C₈HO₂S₂: 2.5 mg; C₉H_11_NO₂: 2.5 mg; C_17_H_20_N_4_O_6_: 2.5 mg; C_10_H_14_N_2_O_3_: 1 mg; C_12_H_17_ClN_4_OS·HCl: 2.5 mg; CHCaN₂O: 2.5 mg; C₈HNO₃·HCl: 5 mg; CHCoNOP: 0.05 mg; and KH₂PO₄: 450 mg), and crude oil was added successively to the soil samples; the whole sample was mixed thoroughly to ensure a uniform distribution of petroleum, and simulated petroleum-contaminated soil with an oil content of 5% was prepared. Sulfate solution (dissolved 1.479 g of Na_2_SO_4_ in 50 mL of H_2_O) was added to the soil samples to form the sulfate-amended group (the sulfate content was set as 1 mg/g dry soil), while the control group was not supplemented with sulfate solution. The soil sample pH was subsequently adjusted to 7, with the gravimetric soil water content regulated to 15% (Wang et al., 2024a). The prepared soil samples were removed and subpackaged in square plastic boxes measuring 13 cm × 13 cm × 8 cm, which were then covered with lids for sealed incubation, to promote the formation of oxygen-limited sealed microcosms within the box. The prepared boxes were placed in constant-temperature incubators at 5 °C, 20 °C, and 40 °C for simulation experiments. Each temperature condition contained a corresponding treatment and control soil sample group. Triplicate replicates were established for every treatment and control group to ensure experimental reproducibility. Soil samples from all the groups were sampled at 7-day intervals. Sterile deionized (DI) water was supplemented weekly after sampling to maintain soil moisture at 15% throughout incubation. Soil sulfate, total nitrogen and available phosphorus concentrations were determined biweekly. The 1 mol/L (NH_4_)_2_SO_4_ solution and 1 mol/L K_2_HPO_4_ solution were added as needed according to the measured values to stabilize the levels of sulfate, total nitrogen and available phosphorus over the entire incubation period. All supplementation solutions were filter-sterilized prior to addition to prevent exogenous microbial contamination (Omenna et al., 2024).

### Extraction and separation of petroleum hydrocarbons

A 9.0 g soil sample was weighed and mixed with anhydrous sodium sulfate at a mass ratio of 1:1 to thoroughly remove free water from the sample. The mixture was placed in a completely dry extraction cup with 60 mL of dichloromethane added, and Soxhlet extraction was performed at 60 °C for 7 h. The entire extract was transferred to a preweighed vial, and the solvent was evaporated to dryness in a fume hood. The vial was reweighed, and the mass difference was calculated as the content of petroleum hydrocarbons (Liu et al., 2020). The petroleum hydrocarbon contents of triplicate samples in each group are presented as the mean ± standard deviation (SD). A three-way full-factorial ANOVA (temperature × sulfate × incubation time) with Type III sum of squares was performed to assess the main effects and all interaction effects among the three experimental factors. Normality and homogeneity of variance were checked by Shapiro–Wilk and Levene tests. Tukey HSD and Welch *t*-tests were used for post-hoc comparisons (*α* = 0.05).

### Fitting of petroleum hydrocarbon degradation kinetics

The weekly measured content of petroleum hydrocarbons was statistically analyzed, and kinetic models were fitted to the degradation data to reveal the temporal trend in petroleum hydrocarbons during degradation. On the basis of the ratio of the remaining petroleum hydrocarbon concentration to the initial concentration (C_t_/C_0_) at different temperatures each week, nonlinear regression analysis of the degradation process was conducted using kinetic models. The goodness of fit of each model was evaluated through the coefficient of determination (*R^2^*), RMSE, AICc, and residual distribution (Karina et al., 2021). Notably, four kinetic models were employed for fitting, and the corresponding half-lives were calculated. The zero-order kinetic model and its half-life are expressed in Equations 1 and 5, respectively, the first-order kinetic model and its half-life are expressed in Equations 2 and 6, respectively, the second-order kinetic model and its half-life are expressed in Equations 3 and 7, respectively, and the pseudo-first-order kinetic model and its half-life are expressed in Equations 4 and 8, respectively (Cabral et al., 2022).


$$C_{t}=-\mathrm{kt}+C_{0}$$ (1)

$$\ln C_{t}=-k_{1}t+\ln C_{0}$$ (2)

$$\begin{array}{c} \frac{1}{C_{t}}=k_{2}t+\frac{1}{C_{0}} \end{array}$$ (3)

$$\begin{array}{c} y=a(1-e^{-k't}) \end{array}$$ (4)

$$\begin{array}{c} t_{\frac{1}{2}}=\frac{C_{0}}{2k} \end{array}$$ (5)

$$\begin{array}{c} t_{\frac{1}{2}}=\frac{\ln2}{k_{1}} \end{array}$$ (6)

$$\begin{array}{c} t_{\frac{1}{2}}=\frac{1}{k_{2}C_{0}} \end{array}$$ (7)

$$\begin{array}{c} t_{\frac{1}{2}}=\frac{1}{k^{\prime }}\ln(\frac{a}{a-0.5}) \end{array}$$ (8)

where C₀ is the initial concentration (mg/g), Cₜ is the pollutant concentration at time t (mg/g), k is the zero-order reaction rate constant (mg·kg^−1^·d^−1^), k_1_ is the first-order reaction rate constant (d^−1^), k₂ is the second-order reaction rate constant (mg^−1^·kg·d^−1^), k^’^ is the first-order reaction rate constant (d^−1^), t is the remediation time (d), y is the degradation rate of petroleum hydrocarbons (%), and a is a constant that can be obtained from the nonlinear regression curve.

### Sample collection for sequencing

At weeks 0, 5, and 9, 1 g of sample was collected from each of the three replicates in every group. Subsamples from the same time point were fully mixed to obtain composite samples characteristic of the initial, intermediate, and late sampling times for subsequent DNA extraction and sequencing (Gou et al., 2022). This pooling strategy was adopted to capture the average community signature of the three triplicate microcosms collected at each sampling time point. The composite samples collected at the 0th, 5th, and 9th weeks of the 5 °C sulfate-amended group were denoted as 5W0E, 5W5E, and 5W9E, respectively, and those of the control group were denoted as 5W0C, 5W5C, and 5W9C, respectively. The samples collected at 0, 5, and 9 weeks in the 20 °C sulfate-amended group were denoted as 20W0E, 20W5E, and 20W9E, respectively, and those in the control group were denoted as 20W0C, 20W5C, and 20W9C, respectively. The samples collected at 0, 5, and 9 weeks in the 40 °C sulfate-amended group were denoted as 40W0E, 40W5E, and 40W9E, respectively, and those in the control group were denoted as 40W0C, 40W5C, and 40W9C, respectively.

### DNA extraction, PCR amplification, and sequencing

Total community genomic DNA was extracted from soil samples with the E. Z. N. A™ Mag-Bind Soil DNA Kit (Omega Bio-Tek, M5635-02, United States) following the manufacturer’s protocols. DNA concentration and purity were assessed on a Qubit 4.0 fluorometer (Thermo Fisher Scientific, United States) to obtain qualified templates for amplicon sequencing (Zhu et al., 2023). The V3–V4 hypervariable region of the bacterial 16S rRNA gene was amplified immediately after DNA extraction using 2 × Hieff® Robust PCR Master Mix (Yeasen Biotechnology, 10105ES03, China) with PAGE-purified universal primers: forward 5′-CCTACGGGNGGCWGCAG-3′ and reverse 5′-GACTACHVGGGTATCTAATCC-3′. The 30 μL reaction mixture contained 2 μL of template DNA (10 ng/μL), 1 μL of each primer (10 μM), and 26 μL of 2 × PCR Master Mix. Amplification was run on an Applied Biosystems 9700 thermal cycler: initial denaturation at 95 °C for 3 min; five cycles (95 °C 30 s, 45 °C 30 s, 72 °C 30 s); 20 cycles (95 °C 30 s, 55 °C 30 s, 72 °C 30 s); and a final extension at 72 °C for 5 min. PCR products were verified by 2% (w/v) TBE-agarose gel electrophoresis with ethidium bromide staining under UV illumination. Amplicons were purified using Hieff NGS™ DNA Selection Beads (Yeasen Biotechnology, 10105ES03, China) to remove free primers and primer dimers and then submitted to Sangon Biotech (Shanghai, China) for library preparation with Illumina adapters and index barcodes. Libraries were quantified via the Qubit® 4.0 dsDNA High-Sensitivity Assay and size-checked on an Agilent 2100 Bioanalyzer (Agilent Technologies, United States). Valid libraries were pooled at equimolar concentrations and subjected to paired-end sequencing on the Illumina MiSeq platform (Illumina, United States) per the manufacturer’s instructions (Cabral et al., 2022).

### OTU clustering, statistical analysis, and function prediction

Raw paired-end Illumina reads were assembled with PEAR (v0.9.8) via sequence overlaps; fastq files were converted into fasta and qual files for downstream analysis. Qualified effective tags were clustered into operational taxonomic units (OTUs) at 97% similarity using USEARCH (v11.0.667). Chimeric sequences and singleton OTUs were removed, and the remaining reads were allocated to the respective samples. The most abundant sequence of each OTU served as the representative sequence, which was taxonomically annotated against the RDP database (Lv et al., 2022). Alpha-diversity indices (Chao 1, Simpson, and Shannon indices) were calculated in Mothur (v3.8.31). Rarefaction and rank-abundance curves were plotted in R (v3.6.0) to assess sequencing adequacy. Moreover, *t*-tests and ANOVA were applied for two-group and multigroup comparisons of alpha-diversity, respectively. The beta-diversity of the bacterial communities was explored using principal coordinate analysis (PCoA). PCoA was performed on the basis of the Bray–Curtis dissimilarities calculated from the OTU relative abundance table to visualize differences in the microbial community composition among the samples. The relative abundances (percentages) of the OTUs were used directly for the computation of Bray–Curtis distances, without Hellinger or other prior transformations. To statistically test the effects of temperature, sulfate, and sampling time on the microbial community composition, a permutational multivariate analysis of variance (PERMANOVA) was applied to the same Bray–Curtis distance matrix. Marginal tests were used, indicating that each term was evaluated after accounting for all the other terms in the model. *p* values were obtained from 9,999 permutations. Ordination analysis was performed using the R vegan package (v2.5–6), and inter-sample distances were visualized in scatter plots. Putative functional profiles were predicted using PICRUSt2 (v2.4.2) from 16S rRNA gene OTU representative sequences. The sequences were inserted into the program’s default reference phylogenetic tree built upon the Integrated Microbial Genomes (IMG) database. Hidden-state prediction was applied under default parameters to estimate gene family copy number abundances, from which MetaCyc pathway abundances were further inferred. The predicted gene family abundances were normalized against 16S rRNA gene copy numbers to account for taxon-specific variation in ribosomal gene copy counts. The nearest-sequenced-taxon index (NSTI) was computed per sample to assess prediction performance (Rong et al., 2023). All community statistical tests were performed on composite pooled sequencing samples, meaning only one sequencing profile was available per treatment at each time point, without independent sequencing replicates corresponding to individual microcosms.

## Results

### Kinetics of petroleum hydrocarbon degradation in soil at different temperatures

The degradation of petroleum hydrocarbons at different temperatures during the remediation process is shown in Figure 1. After the 9th week, the petroleum hydrocarbon degradation efficiencies in the sulfate-amended group were 31.47%, 32.26%, and 35.45% at 5 °C, 20 °C, and 40 °C, respectively, whereas those in the control group were 21.6%, 25.25%, and 27.07%, respectively.

**Figure 1:**
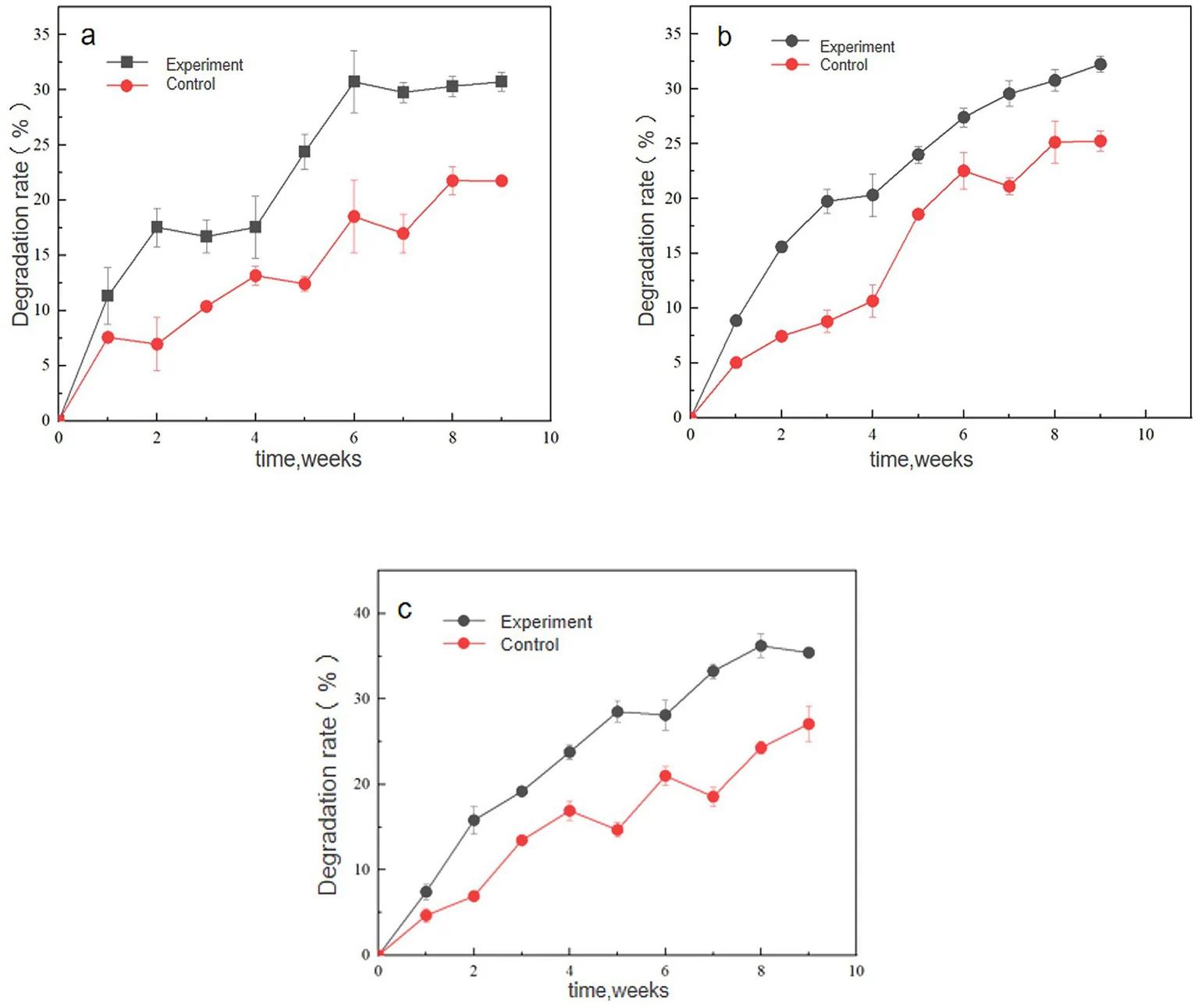
Degradation rate of petroleum hydrocarbons at different temperatures: **(a)** degradation rate of petroleum hydrocarbons at 5 °C; **(b)** degradation rate of petroleum hydrocarbons at 20 °C; and **(c)** degradation rate of petroleum hydrocarbons at 40 °C. Data represent mean ± SD of three biological microcosm replicates.

Three-way full-factorial ANOVA revealed significant main effects of temperature (*F* = 25.35, *p* < 0.001), sulfate amendment (*F* = 1186.29, *p* < 0.001), and incubation time (*F* = 726.21, *p* < 0.001) on petroleum-hydrocarbon degradation efficiency. The two-way interaction between temperature and sulfate was non-significant (*F* = 1.07, *p* = 0.348), indicating that the average magnitude of sulfate-mediated promotion of degradation was comparable across the three incubation temperatures. Significant two-way interactions were detected for temperature × incubation time (*F* = 6.94, *p* < 0.001) and sulfate × incubation time (*F* = 25.34, *p* < 0.001). Furthermore, the three-way interaction temperature × sulfate × incubation time was also significant (*F* = 4.08, *p* < 0.001), demonstrating that the magnitude of the sulfate-driven degradation improvement varied jointly with temperature and incubation time. Pair-wise post-hoc comparisons confirmed that sulfate-amended groups achieved higher degradation efficiency relative to corresponding controls at most incubation time-points across all temperatures.

To predict the petroleum hydrocarbon degradation behavior in the sulfate-amended group at different temperatures, a degradation kinetic model was established. The second-order model exhibited the poorest fitting performance, with *R^2^* values ranging from 0.6413 to 0.6564, indicating its inappropriateness for describing petroleum hydrocarbon degradation in this system. The zero-order and first-order models yielded limited fitting accuracy, with *R^2^* values of 0.7287–0.7472 and 0.8064–0.8261, respectively. In contrast, the pseudo-first-order model produced the optimal fit across all three temperature treatments, with the highest *R^2^* (0.9226–0.9403) and the lowest RMSE and AICc. These findings indicate that the pseudo-first-order kinetic model is more suitable for describing the degradation dynamics of petroleum hydrocarbons in contaminated soil under the three different temperature conditions (Table 2).

**Table 2:** Kinetic parameters of petroleum hydrocarbon degradation in the sulfate-amended group at different temperatures.

Temperature	Kinetic model	*a*	*R^2^*	RMSE	AICc
5 °C	Zero	/	0.7287	2.4706	12.36
	First	/	0.8064	2.0771	8.14
	Second	/	0.6413	3.0025	15.92
	Pseudofirst	0.3402	0.9226	0.0271	−66.31
20°C	Zero	/	0.7395	2.3692	11.74
	First	/	0.8192	1.9643	7.33
	Second	/	0.6508	2.8864	15.11
	Pseudofirst	0.3685	0.9347	0.0239	−68.92
40 °C	Zero	/	0.7472	2.3015	11.22
	First	/	0.8261	1.9054	6.85
	Second	/	0.6564	2.8273	14.70
	Pseudofirst	0.3879	0.9403	0.0222	−70.27

### High-throughput sequencing and microbial community diversity

The numbers of effective sequences and bacterial alpha diversity indices for composite samples at the initial, intermediate, and late sampling times are shown in Table 3. After low-quality reads were removed, 46,138, 54,878, 43,270, 46,480, 51,216, 47,414, 45,880, 42,297, 46,055, 46,171, 43,593, 49,981, 46,296, 51,968, 51,536, 44,927, 41,375, and 52,756 bacterial sequences were obtained from the 5W0C, 5W0E, 5W5C, 5W5E, 5W9C, 5W9E, 20W0C, 20W0E, 20W5C, 20W5E, 20W9C, 20W9E, 40W0C, 40W0E, 40W5C, 40W5E, 40W9C, and 40W9E samples, respectively. The sequencing depth index revealed that the library coverage of the 18 groups of samples exceeded 98%, and the systematic categories of most bacteria in the sampled soil were identified, thus indicating that the sequencing depth was reasonable.

**Table 3:** Effective numbers of sequences and alpha diversity indices of soil microbial communities in different soil samples.

Sample	Effective number	OTUs	Shannon	Chao 1	Ace	Simpson	Coverage
5W0C	46,138	3,259	6.92	3,769.12	3,790.07	0.00	0.98
5W0E	54,878	3,270	6.90	3,679.59	3,718.54	0.00	0.99
5W5C	43,270	3,192	6.94	3,623.54	3,682.46	0.00	0.98
5W5E	46,480	3,218	6.91	3,657.39	3,664.28	0.00	0.99
5W9C	51,216	3,340	6.97	3,729.70	3,771.86	0.00	0.99
5W9E	47,414	3,234	6.97	3,629.04	3,679.34	0.00	0.99
20W0C	45,880	3,284	6.89	3,755.79	3,805.24	0.00	0.98
20W0E	42,297	3,193	6.88	3,648.19	3,706.15	0.00	0.98
20W5C	46,055	3,262	6.83	3,742.09	3,832.61	0.00	0.98
20W5E	46,171	3,183	6.87	3,680.87	3,700.97	0.00	0.98
20W9C	43,593	2,760	5.07	3,340.53	3,368.60	0.09	0.98
20W9E	49,981	3,067	5.94	3,539.34	3599.69	0.03	0.99
40W0C	46,296	3,285	7.00	3,725.68	3,816.32	0.00	0.98
40W0E	51,968	3,400	7.01	3,819.97	3,839.20	0.00	0.99
40W5C	51,536	3,140	6.48	3,560.23	3,589.62	0.01	0.99
40W5E	44,927	3,127	6.71	3,598.80	3,647.56	0.01	0.98
40W9C	41,375	3,100	6.88	3,513.61	3,596.79	0.00	0.98
40W9E	52,756	3,129	6.88	3,511.89	3,510.01	0.00	0.99

The sequences were clustered into OTUs, and the number of OTUs across the 18 sample groups ranged from 2,760 to 3,400 (all the detailed data are listed in Supplementary Table S1). Soil bacterial alpha diversity indices were further calculated. On the basis of the Shannon, Chao 1, and ACE metrics, at the initial sampling time of 40 °C, the overall microbial diversity was very high, and the diversity of the sulfate-amended group composite sample was greater than that of the control group composite sample; nevertheless, the diversity gradually converged at later sampling times. At 20 °C, high diversity was observed in both group composite samples at the initial sampling times and persisted until the intermediate sampling times. At the late sampling time, the composite sample of the control group showed reduced community richness and evenness. A decline in diversity was also observed in the sulfate-amended composite sample, though the magnitude of change appeared smaller relative to the control. At 5 °C, all the alpha diversity parameters of the sulfate-amended and control groups composite samples remained high, with only negligible fluctuations throughout the entire degradation cycle. These results suggest that temperature variation and sulfate addition jointly shape soil microbial diversity during the degradation process.

PERMANOVA based on Bray–Curtis dissimilarities revealed that both the incubation temperature and the remediation stage significantly shaped the microbial community structure (Table 4). Temperature was the dominant factor (pseudo-*F*: 4.800; *R^2^*: 0.3603; *p*: 0.0001), explaining approximately 36.0% of the total variation. The remediation stage also had a significant effect (pseudo-*F*: 2.164, *R^2^*: 0.1624, *p*: 0.0130), accounting for 16.2% of the variation. In contrast, sulfate amendment did not significantly affect the community composition (pseudo-*F*: 0.717; *R^2^*: 0.0269; *p*: 0.6771), contributing only 2.7% of the variation. Consistent with the PERMANOVA results, the PCoA ordination (Figure 2) provided visual support for the effects of temperature, sulfate, and stage. The first two principal coordinates explained 11.69% (PCoA1) and 8.81% (PCoA2) of the total variation, respectively. A clear temperature gradient was evident along PCoA1: the 5 °C groups clustered toward the positive end of PCoA1, the 40 °C groups shifted toward the negative end, and the 20 °C groups occupied an intermediate position. This axis-level separation among temperature groups is consistent with the highly significant temperature effect.

**Table 4:** PERMANOVA results based on Bray–Curtis dissimilarities.

Term	df	SS	pseudo-*F*	*R^2^*	*p* (>*F*)
Temperature	2	0.28412	4.800	0.3603	0.0001
Sulfate	1	0.02123	0.717	0.0269	0.6771
Stage	2	0.12807	2.164	0.1624	0.0130
Residual	12	0.35513	–	0.4504	–
Total	17	0.78855	–	1.000	–

**Figure 2:**
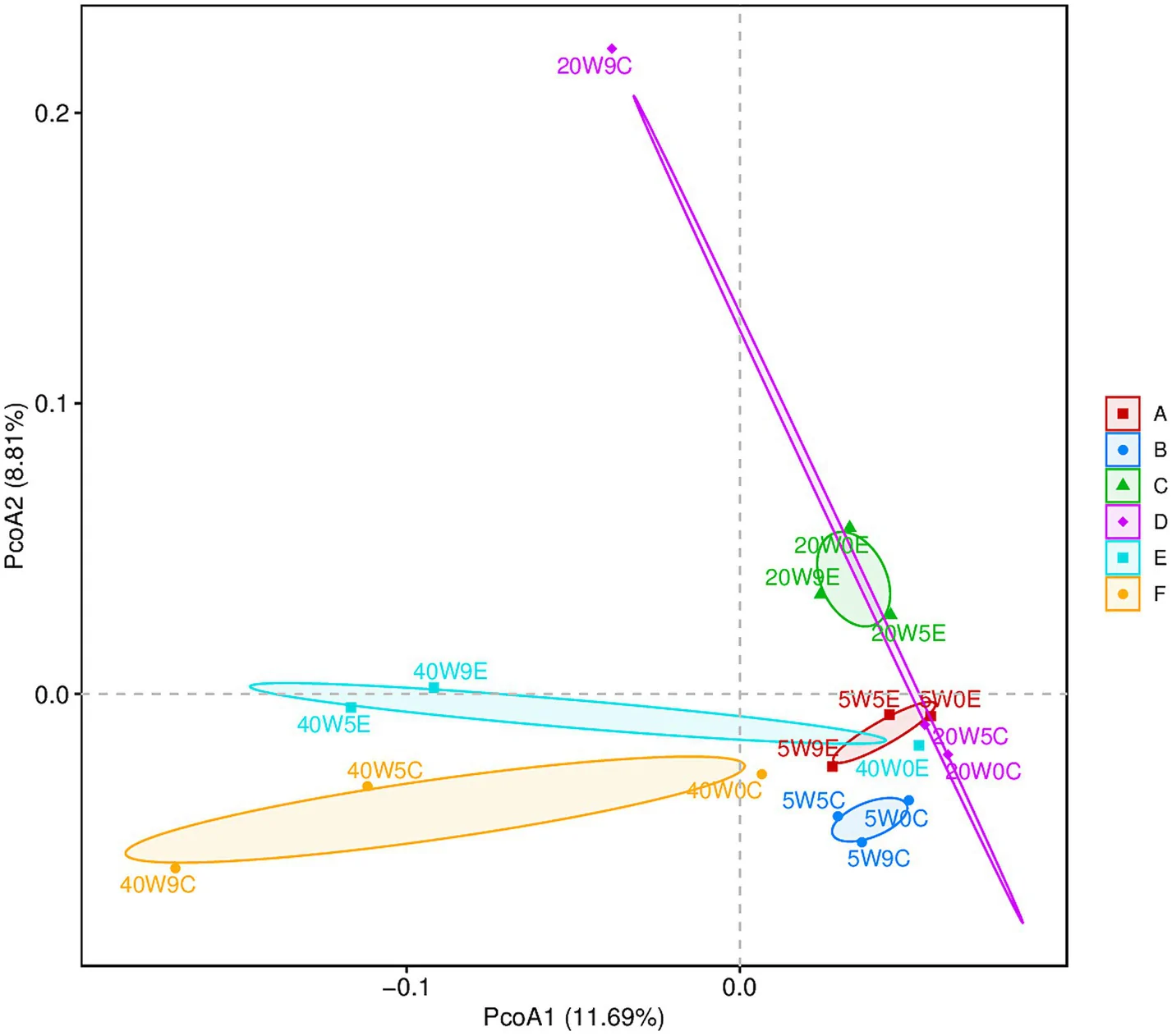
Principal coordinate analysis diagram (Groups A, C, and E represent the sulfate-amended group composite samples at 5 °C, 20 °C, and 40 °C, respectively; Groups B, D, and F represent the control group composite samples at 5 °C, 20 °C, and 40 °C, respectively).

### Microbial community composition

The relative abundance distribution of microbial communities at different sulfate concentrations during different degradation periods is shown in Figure 3.

**Figure 3:**
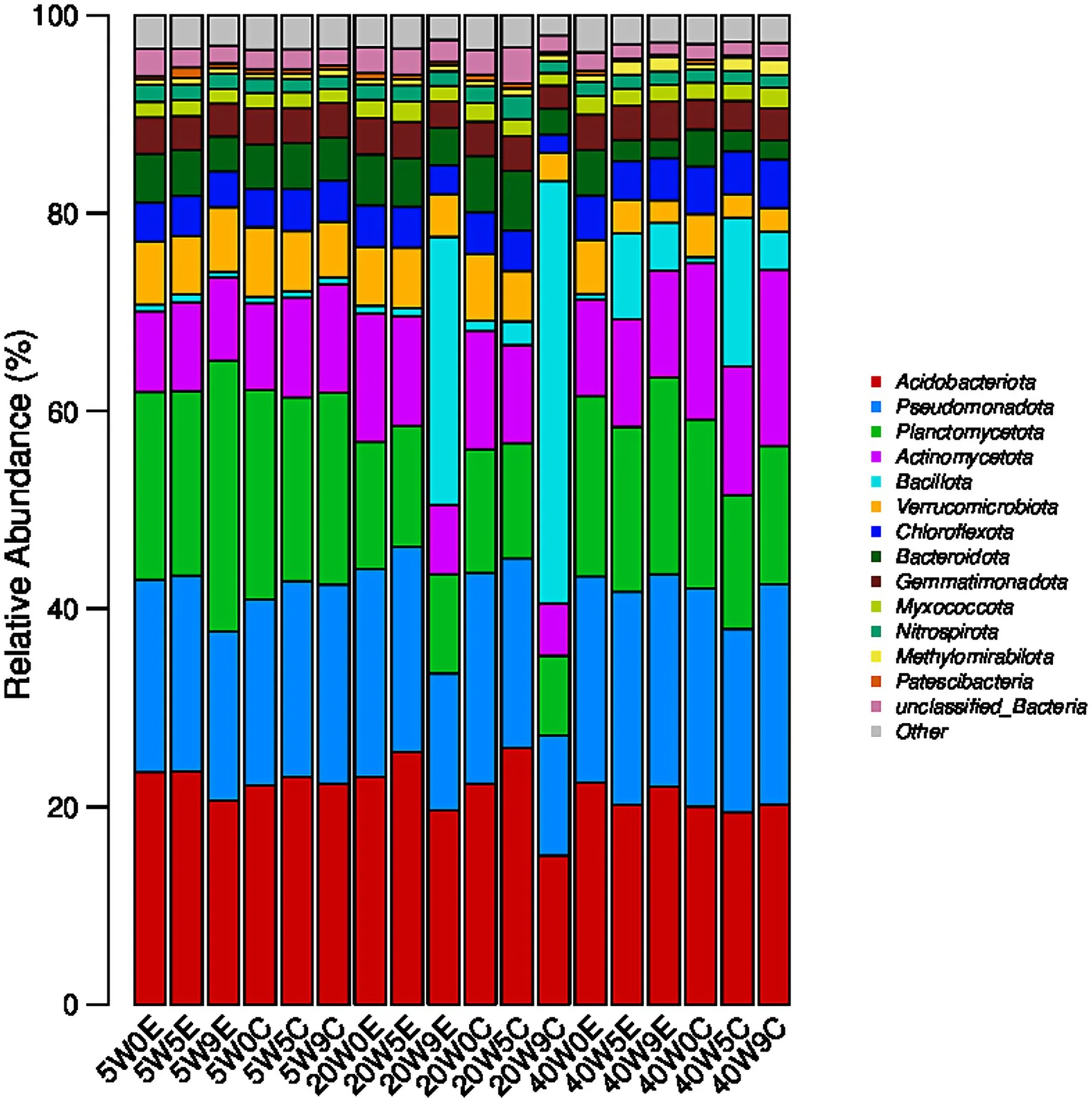
Illumina composition of different microbial community structures obtained from sequencing (E represents the sulfate-amended group composite sample, and C represents the control group composite sample). 0, 5, and 9 refer to the 0th, 5th, and 9th weeks, respectively.

An analysis of the 5 °C environment revealed no obvious change in the microbial community in either the treatment or the control group composite samples. The relative abundance levels of *Pseudomonadota* (19.46–19.82%), *Acidobacteriota* (23.48–23.54%), and *Actinomycetota* (8.13–8.99%) remained relatively stable at the initial and intermediate sampling times. At the late sampling time, the relative abundance of the dominant phyla in the soil changed, and the relative abundance of *Planctomycetota* increased (18.65% → 27.46%), thus indicating that it became the dominant microbial community in the soil.

An analysis of the 20 °C environment revealed that the relative abundance of *Acidobacteriota* increased (22.98% → 25.49%), whereas the relative abundance of *Pseudomonadota* remained relatively stable (21.01% → 20.72%) and dominated at this stage. At the intermediate and late sampling times, the relative abundance of *Bacillota* sharply increased to 27.09%, whereas the relative abundance levels of the other phyla decreased, indicating that *Bacillota* became the dominant phylum.

Analysis of the 40 °C treatment revealed that the microbial community was consistently dominated by *Pseudomonadota*, *Actinomycetota*, *Bacillota*, and *Planctomycetota* throughout the entire degradation period, with these phyla sustaining their predominance across all incubation stages.

### Prediction of microbial functions under sulfate stimulation

The weighted NSTI values of all the samples ranged from 0.26 to 0.31 (mean 0.29), well above the recommended threshold of 0.15, indicating that a considerable proportion of the soil microbial community lacked closely related reference genomes. Accordingly, the functional profiles predicted by PICRUSt2 should be interpreted as indicating a putative metabolic potential rather than direct evidence of *in situ* activity. Despite this limitation, the heatmap (Figure 4) revealed differential enrichment of KO gene families across treatments. Gene families related to fatty acid metabolism (fabG, fabF, atoB, and fadD), ABC-type transport systems (ABC. PE and ABC. FEV), and stress-responsive sigma factors (rpoE) tended to be enriched under higher temperature or prolonged incubation, which coincided with the taxonomic shifts observed at the phylum level (Figure 3).

**Figure 4:**
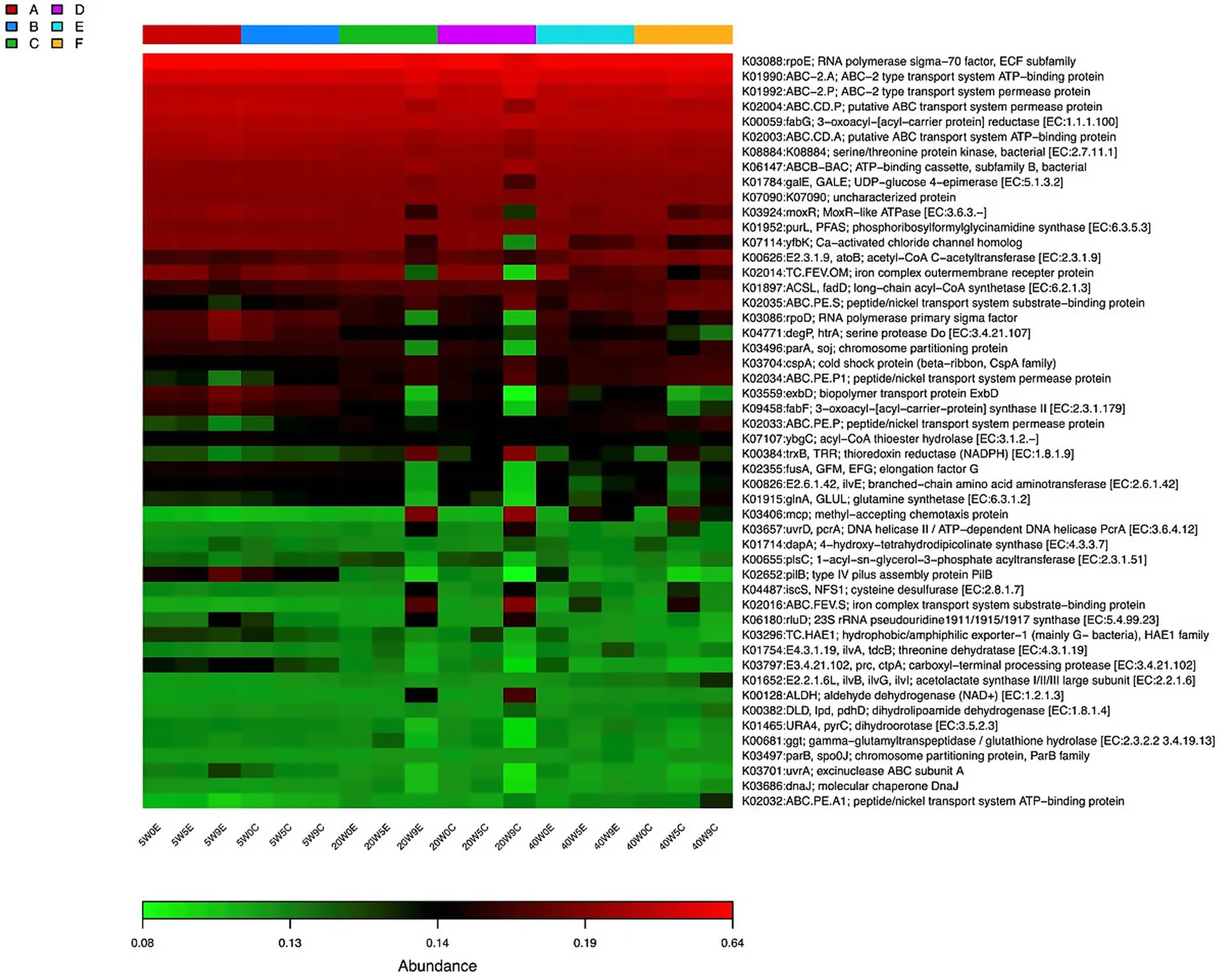
KEGG tertiary pathways of soil bacteria under different experimental methods (top 50; A and B refer to the treatment and control group composite samples, respectively, at 5 °C; C and D refer to the treatment and control group composite samples, respectively, at 20 °C; E and F refer to the treatment and control group composite samples, respectively, at 40 °C).

## Discussion

### Degradation of Petroleum Hydrocarbon

In accordance with the characteristics of the pseudo-first-order kinetic model, petroleum hydrocarbon degradation under all three temperature treatment groups exhibited a rapid degradation rate at the initial stage, followed by a decreased degradation rate in the intermediate and late stages. This phenomenon may be attributed to the rapid consumption of readily degradable petroleum hydrocarbon fractions in the initial phase. As recalcitrant fractions became dominant, the degradation rate gradually decreased. In addition, the adsorption and sequestration of petroleum hydrocarbons by soil particles can reduce pollutant bioavailability, which also contributes to the limitation of degradation rates. As the incubation temperature increased from 5 °C to 40 °C, the theoretical maximum degradation ratio a of the pseudo-first-order model increased from 0.3402 to 0.3879, suggesting that 40 °C resulted in the highest degradation among the temperatures tested. Since the fitted values were less than 0.5 for all the temperature groups, the actual petroleum hydrocarbon degradation half-life could not be calculated from the treatment data, which implied that a considerable fraction of petroleum hydrocarbons were resistant to microbial degradation in the soil.

The soil petroleum hydrocarbon degradation efficiency varied significantly with temperature. In the sulfate-amended groups, compared with week 0, the degradation efficiencies at 5, 20, and 40 °C reached 25.0%, 26.4%, and 28.51% by week 5, respectively, and then further increased to 31.47%, 32.26%, and 35.45% by week 9, respectively, demonstrating a positive correlation between temperature and degradation efficiency at the tested temperatures. This positive effect can be attributed to the fact that elevated temperature enhances the activity of degrading enzymes and promotes the dominance of efficient hydrocarbon-degrading microbial consortia (An et al., 2023). Furthermore, the degradation efficiencies in the control groups were consistently lower than those in the sulfate-amended groups across all the sampling periods, indicating that sulfate amendment facilitated the degradation of petroleum hydrocarbons in the contaminated soil. This increase is probably because, in oxygen-limited microcosms, sulfate serves as an adequate terminal electron acceptor for sulfate-reducing bacteria, stimulating their growth and thereby sustaining the degradation of hydrocarbons (Beilig et al., 2024).

### Effects of sulfate amendment on the microbial community at 5 °C

In the 5 °C sulfate-amended group, distinct compositional shifts were detected in the microbial community across sequential degradation stages. The initial degradation community was dominated by *Pseudomonadota* and *Acidobacteriota*. From the intermediate sampling times onward, the relative abundance of *Acidobacteriota* gradually decreased, whereas the proportion of *Planctomycetota* increased continuously, driving a directional shift in community composition. This temporal pattern is consistent with the differing substrate utilization preferences of these two phyla reported in prior research (Yong-Rui and Mu-Tai, 2022; Zhiying et al., 2022): petroleum hydrocarbons are progressively decomposed into low-molecular-weight substrates during incubation, a substrate pool that may favor taxa affiliated with *Planctomycetota* rather than *Acidobacteriota*. In the late degradation phase, the relative abundance of *Planctomycetota* sharply increased. This succession trend may be associated with two co-occurring environmental changes in the 5 °C microcosms: progressive depletion of labile hydrocarbon substrates and persistent low-temperature conditions. As a typical cold-adapted oligotrophic phylum, *Planctomycetota* appears better suited to thrive under these late-stage limiting conditions, which potentially contributes to its eventual predominance (Bayatian et al., 2025).

### Effects of sulfate amendment on the microbial community at 20 °C

At the initial sampling times, the microbial community was dominated by *Pseudomonadota* and *Acidobacteriota*, which is consistent with the community composition observed under the 5 °C treatment. However, a clear distinction emerged under 20 °C incubation: the relative abundance of *Acidobacteriota* increased continuously from the initial to the intermediate degradation phase. This temporal trend is consistent with temperature-driven shifts in soil hydrocarbon retention; elevated temperature may reduce soil adsorption of petroleum substrates, which potentially facilitates substrate acquisition by taxa within *Acidobacteriota* (Chen et al., *2025*). At late sampling times, the relative abundance of *Bacillota* sharply increased. Previous studies have reported that members affiliated with *Bacillota* can utilize recalcitrant asphaltenes, resins, and intermediate metabolites generated during initial hydrocarbon transformation. This documented physiological trait aligns with the observed late-stage enrichment of *Bacillota* (Ida et al., 2021).

### Effects of sulfate amendment on the microbial community at 40 °C

In the 40 °C sulfate-amended group, *Pseudomonadota*, *Actinomycetota*, *Bacillota*, and *Planctomycetota* remained the dominant phyla throughout the entire degradation process, with their relative abundances maintaining relatively stable temporal profiles. This stable community structure may be associated with high-temperature-induced changes in soil hydrocarbon properties. Specifically, incubation at 40 °C potentially increases the fluidity of petroleum hydrocarbons, which could improve substrate accessibility for indigenous microorganisms (Tj et al., 2023; Tijana et al., 2022). Furthermore, moderately high temperatures may support enhanced microbial metabolic activity, which is consistent with the sustained hydrocarbon degradation performance observed under high-temperature conditions (Bonggyu et al., 2020; Wang et al., 2024b).

### Effects of sulfate amendment on microbially mediated hydrocarbon degradation

At 5 °C, the sulfate-amended and control microcosms were initially codominated by *Acidobacteriota* and *Pseudomonadota*. The sulfate-amended group exhibited more pronounced community succession: the relative abundance of *Acidobacteriota* decreased continuously during the incubation period, whereas that of *Planctomycetota* markedly increased during the late sampling period. The control group showed milder compositional shifts and sustained higher proportions of *Acidobacteriota* and *Pseudomonadota*. At 20 °C, the initial-stage community structure was comparable between the sulfate-amended and control treatments. Within sulfate-amended microcosms, *Acidobacteriota* accumulated from initial to mid-term degradation, followed by notable enrichment of *Bacillota* at the late phase. In contrast, *Bacillota* remained rare in the nonsulfate controls, where *Acidobacteriota* and *Planctomycetota* remained as the dominant phyla throughout the incubation period. At 40 °C, *Pseudomonadota*, *Actinomycetota*, *Bacillota*, and *Planctomycetota* dominated both groups across all the sampling time points, with only slight temporal fluctuations in their relative abundances. Compared with the control group, the sulfate-amended group maintained consistently higher proportions of *Bacillota*. Unlike the 5 °C and 20 °C systems, no dramatic turnover of dominant taxa was observed in either treatment at 40 °C.

### Limitations of this study

In this study, all community ordination and PERMANOVA results reflect compositional differences between pooled composite samples rather than independent biological replicates of microcosms. Furthermore, all associations drawn between bacterial community shifts, sulfur cycling and anaerobic hydrocarbon transformation remain speculative, as they are solely inferred from 16S rRNA taxonomic profiles and previous published studies, without supporting in-situ metabolic measurement data.

## Conclusion

In this study, we investigated the effects of sulfate, which serves as a terminal electron acceptor, on petroleum hydrocarbon biodegradation and microbial community dynamics in contaminated soils in oxygen-limited microcosms across different temperature regimes. Sulfate amendment significantly improved hydrocarbon degradation efficiency; however, the extent of this beneficial effect was modulated jointly by temperature and incubation time. Degradation kinetic modeling demonstrated that the pseudo-first-order model effectively described the degradation processes at 5 °C, 20 °C, and 40 °C. Gene families associated with fatty acid metabolism, ABC-type transport systems, and stress-responsive sigma factors tended to be enriched under elevated-temperature conditions or with extended incubation time. Temperature was the main factor structuring community composition, whereas the overall effect of sulfate amendment on community composition was weak and not statistically significant.

## Data Availability

The raw sequencing data of this study have been deposited in the NCBI Sequence Read Archive (SRA) (https://www.ncbi.nlm.nih.gov/sra) under BioProject accession PRJNA1542268. The SRA run accessions include SRR11386120, SRR11386091, SRR11386089, SRR11386064, SRR11386119, SRR11386118, SRR11386121, SRR11386122, SRR11386065, SRR11386090, SRR11386088, SRR11386063, SRR11386139, SRR11386140, SRR11386141, SRR11386137, SRR11386135, SRR11386136.
